# Comparative Efficacy and Functional Outcomes of Psychedelic-Assisted Therapies in Treatment-Resistant Depression: A Systematic Review of Recent Clinical Trials

**DOI:** 10.7759/cureus.82532

**Published:** 2025-04-18

**Authors:** Ciara Mimms, Kassandra Sotelo, Abdul Saboor Khaliq

**Affiliations:** 1 Internal Medicine and Psychiatry, St. George's University School of Medicine, West Indies, GRD; 2 Internal Medicine, Allama Iqbal Medical College, Lahore, PAK

**Keywords:** antidepressant response, cognitive effects, depression scales, ketamine, psilocybin, psychedelic-assisted therapy, psychiatry, randomized controlled trial, systematic review, treatment-resistant depression

## Abstract

This systematic review explores the comparative efficacy and functional outcomes of psychedelic-assisted therapies in the management of treatment-resistant depression (TRD). Following the Preferred Reporting Items for Systematic Reviews and Meta-Analyses (PRISMA) guidelines, a comprehensive literature search was conducted across PubMed, Scopus, and Web of Science for randomized controlled trials (RCTs) published in the last 12 months. Ten RCTs were included, evaluating agents such as ketamine, esketamine, and psilocybin. Most studies demonstrated significant reductions in depressive symptom severity, with oral and intranasal esketamine and high-dose psilocybin showing sustained antidepressant effects. Functional improvements, such as workplace productivity and cognitive stability, were reported in select trials, notably those involving esketamine. Risk of bias was low in four studies and moderate in six due to open-label or observational extensions. Overall, psychedelic therapies were well tolerated, with favorable safety profiles and minimal cognitive adverse effects. These findings support the integration of psychedelic-assisted therapies as viable alternatives or adjuncts in the treatment of TRD and highlight the importance of assessing both clinical and functional endpoints for a more holistic understanding of therapeutic benefit.

## Introduction and background

Treatment-resistant depression (TRD) poses a significant global health burden, affecting an estimated 30% of individuals with major depressive disorder who fail to respond to at least two adequate trials of antidepressant therapies administered at appropriate doses and durations-typically six to eight weeks - as defined by standard guidelines, such as those of the American Psychiatric Association (APA) [1]. Conventional treatment modalities, including pharmacotherapy, psychotherapy, and electroconvulsive therapy (ECT), often yield limited results in this population, prompting the urgent need for novel and more effective interventions [2,3]. In recent years, psychedelic-assisted therapies - particularly those involving ketamine, esketamine, psilocybin, and other serotonergic or glutamatergic agents - have emerged as promising alternatives for individuals with TRD [4]. These therapies work through mechanisms distinct from traditional antidepressants, including rapid modulation of neuroplasticity, enhancement of functional brain connectivity, and disruption of maladaptive cognitive patterns [4].

Ketamine and its S-enantiomer, esketamine, have shown rapid antidepressant effects and are clinically approved for use in several countries. Their inclusion in standard clinical guidelines, such as the Maudsley Prescribing Guidelines, 15th Edition, further supports their legitimacy as established treatment options in the management of TRD. Meanwhile, psilocybin-assisted psychotherapy is gaining momentum through ongoing clinical trials [5]. However, while efficacy data are encouraging, it is equally crucial to evaluate the functional outcomes of these therapies (e.g., improvements in cognitive performance, daily functioning, work productivity, and overall quality of life), which directly influence long-term recovery and reintegration into society [6].

Despite the growing body of clinical trials, comparative studies that evaluate both efficacy and functional impact across different psychedelic-assisted therapies remain sparse. Existing reviews tend to focus narrowly on symptom reduction without capturing the broader therapeutic value or long-term benefits of these interventions. Therefore, this systematic review aims to synthesize and compare the current evidence on the efficacy and functional outcomes of various psychedelic-assisted therapies in patients with TRD. By analyzing clinical trials involving psilocybin, ketamine, esketamine, and related compounds, the study seeks to provide a comprehensive perspective on their therapeutic potential beyond symptomatic relief.

This systematic review sought to assess the comparative efficacy and functional outcomes of psychedelic-assisted therapies in the management of TRD. The population [7] includes adult patients diagnosed with TRD who have not achieved an adequate response with at least two prior antidepressant treatments. The interventions of interest are psychedelic-assisted therapies, including but not limited to intravenous or oral ketamine, intranasal esketamine, psilocybin-assisted psychotherapy, or related compounds.

The comparators may include placebo, standard antidepressant therapy, ECT, or other psychedelic agents. The outcomes evaluated are both clinical (e.g., response rate, remission, reduction in depressive symptoms) and functional (e.g., cognitive performance, occupational functioning, daily living activities, quality of life). This review seeks to determine which psychedelic therapies offer the most robust improvements in both symptom severity and functional well-being in patients with TRD.

## Review

Materials and methods

Search Strategy

The literature search was conducted in accordance with the Preferred Reporting Items for Systematic Reviews and Meta-Analyses (PRISMA) guidelines [8] to ensure methodological rigor, transparency, and reproducibility. A comprehensive search strategy was implemented across PubMed, Scopus, and Web of Science to identify relevant randomized controlled trials (RCTs) evaluating the efficacy and functional outcomes of psychedelic-assisted therapies in TRD. The PubMed search included a combination of keywords and MeSH terms, such as “treatment-resistant depression” OR “TRD” AND “psilocybin” OR “ketamine” OR “esketamine” OR “psychedelic therapy” AND “randomized controlled trial” OR “RCT”, with filters applied for human studies and publications from the last 12 months. Only RCTs involving human participants, published in English within the past year, were considered eligible. Studies were screened based on predefined inclusion and exclusion criteria, focusing on interventions involving ketamine, esketamine, psilocybin, and related compounds. Titles and abstracts were independently reviewed, followed by full-text screening. Priority was given to studies reporting both clinical efficacy (e.g., Montgomery-Åsberg Depression Rating Scale (MADRS), Hamilton Depression Rating Scale (HDRS), Quick Inventory of Depressive Symptomatology (16-item)-Self-Report (QIDS-SR16)) and functional or cognitive outcomes to provide a comprehensive assessment of therapeutic impact.

Eligibility Criteria

Studies were selected based on a predefined set of inclusion and exclusion criteria designed to ensure relevance, quality, and applicability to the research objective. Eligible studies included RCTs published in peer-reviewed journals within the last 12 months (April 2024-April 2025), written in English, and involving human participants diagnosed with TRD. Interventions of interest were psychedelic-assisted therapies, including but not limited to ketamine (intravenous, oral, or intranasal), esketamine, psilocybin, and other emerging psychedelic compounds administered with or without adjunctive psychotherapy. Comparators included placebo, standard antidepressants, electroconvulsive therapy (ECT), or other active control treatments.

To be included, studies were required to report on clinical efficacy outcomes using standardized depression rating scales [9] (e.g., MADRS, HDRS, QIDS-SR16) and, where available, functional or cognitive outcomes, such as workplace productivity, functional remission (e.g., Sheehan Disability Scale), or neurocognitive performance. Exclusion criteria encompassed non-randomized studies, case reports, editorials, reviews, animal studies, and articles lacking sufficient data on treatment efficacy or functional impact. Studies with a primary focus on populations with psychotic disorders, bipolar depression, or non-TRD diagnoses were also excluded to maintain population specificity and clinical relevance.

Data Extraction

A standardized data extraction form was developed to ensure consistency and accuracy in capturing key information from each included study. Two reviewers independently extracted data on study characteristics (author, year, sample size, country), participant demographics (age, gender, diagnostic criteria), intervention details (psychedelic agent, dosage, route of administration, frequency, and duration), comparator arms, outcome measures, and main findings. Specific attention was given to clinical efficacy outcomes measured using standardized depression scales (e.g., MADRS, HDRS, QIDS-SR), as well as any reported functional or cognitive outcomes, such as productivity indices, disability scales, or cognitive test batteries. Adverse events and safety profiles were also extracted where available. Discrepancies between reviewers were resolved through discussion and, when necessary, consultation with a third reviewer.

Data Analysis and Synthesis

Given the heterogeneity of interventions, outcome measures, and reporting styles across studies, a narrative synthesis approach was adopted for data analysis. Findings were organized thematically based on intervention type (e.g., ketamine, esketamine, psilocybin), primary outcomes (e.g., symptom reduction, relapse prevention), and functional or cognitive domains. Where applicable, comparative efficacy was highlighted using reported statistical values, such as mean score changes, p-values, and correlation coefficients. Functional outcomes, when available, were synthesized to complement the clinical findings and provide a holistic understanding of treatment impact. Risk of bias for each study was assessed using the Cochrane Risk of Bias 2 (RoB 2) tool [10], and results were tabulated to support the qualitative synthesis. Meta-analysis was not performed due to variability in trial design and outcome reporting, but the narrative approach allowed for a robust comparative evaluation across interventions.

Results

Study Selection Process

**Figure 1:**
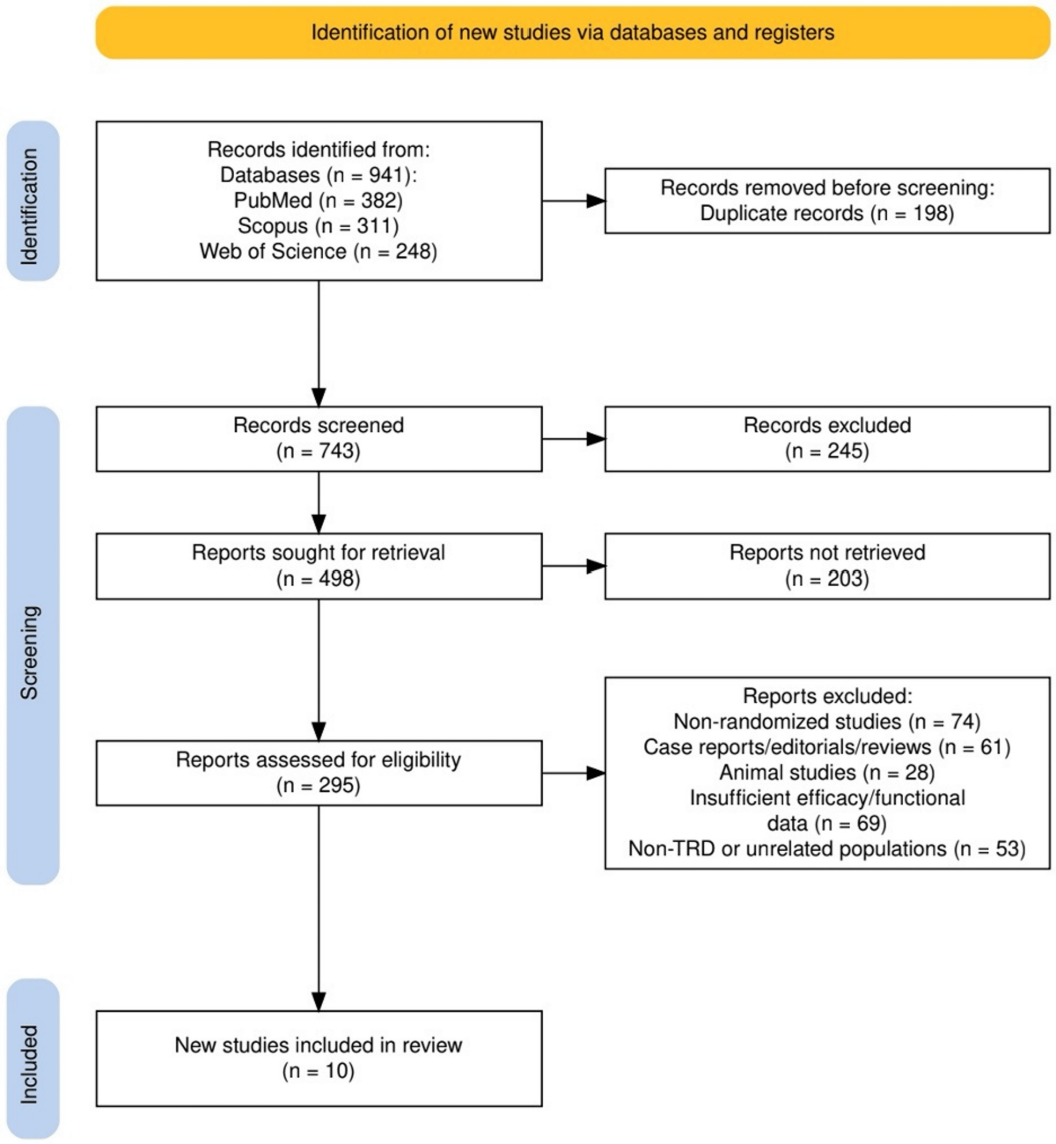
The PRISMA flowchart represents the study selection process. PRISMA: Preferred Reporting Items for Systematic Reviews and Meta-Analyses

The study selection process followed the PRISMA 2020 guidelines, as illustrated in Figure 1. A total of 941 records were initially identified through database searches - PubMed (n = 382), Scopus (n = 311), and Web of Science (n = 248). After removing 198 duplicate records, 743 records remained for title and abstract screening. Of these, 245 were excluded based on irrelevance to the research question; 498 full-text reports were sought for retrieval, of which 203 could not be retrieved, leaving 295 reports for full-text assessment. Following detailed evaluation, 285 studies were excluded for reasons including being non-randomized trials (n = 74), case reports or reviews (n = 61), animal studies (n = 28), insufficient data on efficacy or functional outcomes (n = 69), or focus on non-TRD populations (n = 53). Ultimately, 10 studies met the eligibility criteria and were included in the final review.

Characteristics of the Selected Studies

As presented in Table 1, the 10 included studies were all RCTs published between 2024 and 2025, collectively encompassing a diverse sample of adults diagnosed with TRD. The interventions evaluated consisted of various psychedelic-assisted therapies, including oral, intravenous, and intranasal ketamine/esketamine, psilocybin, and augmentation strategies such as rTMS or aripiprazole. Sample sizes ranged from 61 to 884 participants, with comparators including placebo, ECT, standard antidepressants, or active controls, such as midazolam and quetiapine. Across the studies, clinical efficacy was predominantly measured using validated depression rating scales, such as the MADRS, HDRS, QIDS-SR16, and Dossier Santé Québec (DSQ). Several trials also incorporated functional and cognitive outcomes, including the Sheehan Disability Scale, workplace productivity measures, and cognitive batteries. Most interventions demonstrated statistically significant reductions in depressive symptoms, while some, notably esketamine and psilocybin, showed potential in extending remission duration and enhancing real-world functioning. Safety profiles were generally favorable, with minimal cognitive adverse effects reported and tolerability rated as good across multiple settings.

**Table 1:** A summary of all the selected studies in the systematic review. TRD – Treatment-Resistant Depression; RCT – Randomized Controlled Trial; AE – Adverse Event; NS – Nasal Spray; SC – Subcutaneous; TID – Ter In Die (Three Times a Day); IV – Intravenous; HRSD – Hamilton Rating Scale for Depression; HDRS17 – 17-item Hamilton Depression Rating Scale; BDI – Beck Depression Inventory; CGI – Clinical Global Impression; SDS – Sheehan Disability Scale; WPAI:D – Work Productivity and Activity Impairment Questionnaire: Depression; QIDS-SR16 – Quick Inventory of Depressive Symptomatology - Self Report, 16 items; DSQ – Depression Symptom Questionnaire; MADRS – Montgomery–Åsberg Depression Rating Scale; HVLT-R – Hopkins Verbal Learning Test-Revised; 5D-ASC – 5-Dimensional Altered States of Consciousness Rating Scale; R-107 – Research Code for Oral Ketamine Formulation; ECT – Electroconvulsive Therapy; rTMS – Repetitive Transcranial Magnetic Stimulation; SSRI – Selective Serotonin Reuptake Inhibitor; SNRI – Serotonin-Norepinephrine Reuptake Inhibitor; XR – Extended Release; COMP360 – Psilocybin Formulation by COMPASS Pathways

Study	Population	Intervention	Comparator	Outcomes Measured	Key Results	Functional Outcomes	Adverse Events/Safety	Conclusion
Glue et al., 2024 [11]	Adults with TRD (n = 168 randomized after enrichment)	Oral ketamine tablet (R-107), 30–180 mg, twice weekly	Placebo	MADRS score change at 13 weeks; response/relapse rates	180 mg group: MADRS ↓ by 6.1 vs placebo (P = 0.019); relapse: 43% vs 71%	No formal functional outcomes; home-based dosing supports real-world feasibility	Excellent tolerability; minimal sedation or BP changes	Oral ketamine effective and well-tolerated for TRD
Jha et al., 2024 [12]	Adults (n = 365); nonpsychotic TRD	IV ketamine (6 infusions over 3 weeks)	ECT (9 sessions over 3 weeks)	QIDS-SR16 & MADRS; subgroup analysis by severity, inpatient/outpatient	Ketamine > ECT in moderate outpatient TRD; ECT > ketamine early in severe inpatient cases	No formal functional outcomes; indirect insights via cognitive and subgroup data	Safety not detailed in this analysis	Ketamine preferable for outpatient moderate TRD; ECT for severe inpatient cases
Papakostas et al., 2024 [13]	Adults with TRD (n = 278)	rTMS or aripiprazole augmentation	Switch to venlafaxine/duloxetine	MADRS & Depression Symptom Questionnaire (DSQ)	rTMS > switch on MADRS (P = 0.015); aripiprazole > switch on DSQ (P = 0.003)	No direct functional outcomes; symptom-based measures only	Not detailed in abstract	rTMS more effective than switching; supports augmentation strategy
Goodwin et al., 2025 [14]	Adults with TRD (n = 233); COMP360 psilocybin	25 mg, 10 mg, or 1 mg psilocybin + therapy	Dose comparisons	MADRS; 5D-ASC & Emotional Breakthrough Inventory	At 25 mg, strong correlation between intensity of psychedelic experience & MADRS ↓ (r = -0.51 to -0.64)	No direct functional data; psychedelic experience linked to outcomes	Not detailed in abstract	25 mg psilocybin’s effects tied to subjective psychedelic experience
Smith-Apeldoorn et al., 2024 [15]	Adults with TRD (n = 111)	Oral esketamine 30 mg TID (6 weeks), open-label titrated (0.5–3.0 mg/kg)	Placebo during fixed-dose phase	HDRS17 change at 6 weeks and in open-label phase	No benefit at fixed dose; titrated phase: HDRS ↓ by 6 points (P < 0.001)	Functional outcomes not assessed	Dizziness, sleep hallucinations ↑ with esketamine; generally safe	Titrated oral esketamine may be effective; fixed dose not
Martin et al., 2024 [16]	Adults with TRD (n = 174); KADS trial	SC ketamine (fixed or escalating dose)	Active control: midazolam	Cognitive performance from baseline to end	No significant cognitive differences between ketamine and control	No change in cognitive function; supports cognitive safety	No major cognitive AEs reported	Repeated ketamine did not impair cognition in short term
Morrison et al., 2024 [17]	Adults with TRD (n = 884 across 4 RCTs)	Esketamine nasal spray (28–84 mg) + oral antidepressant	Placebo nasal spray + oral antidepressant	Cogstate battery & HVLT-R; MADRS correlations	Cognitive performance stable or improved; no group differences	Cognition preserved; mild baseline impairments improved over time	No cognitive safety concerns identified	Esketamine safe for cognition in both short- and long-term use
Vieta et al., 2025 [18]	Adults with TRD (n = 676); ESCAPE-TRD trial	Esketamine NS + SSRI/SNRI	Quetiapine XR + SSRI/SNRI	SDS for functioning; WPAI:D for productivity	Functional remission weeks: +43.2% with esketamine (P = 0.0023); productivity loss ↓ (P = 0.0045)	Strong improvement in functioning, absenteeism, presenteeism	Not detailed; tolerability assumed comparable	Esketamine NS improved function and productivity vs quetiapine
Goodwin et al., 2025 [19]	TRD patients from COMP001/003 (n = 66 in follow-up)	25 mg COMP360 psilocybin (1 dose)	10 mg and 1 mg COMP360	Time to depressive relapse over 52 weeks	Median time to depressive event: 25 mg = 189 days; 10 mg = 43 days; 1 mg = 21 days (post hoc)	No functional measures; longer remission suggests life impact	Few AEs; 1 suicidal ideation in 1 mg group	25 mg psilocybin shows longer antidepressant durability
Kumar et al., 2024 [20]	Adults with TRD (n = 61); outpatients	Oral ketamine 150 mg (7 sessions)	IV ketamine 0.5 mg/kg (7 sessions)	HRSD, MADRS, BDI, CGI at days 14 & 30	No efficacy difference; oral ketamine had lower dropout(26.7% vs 54.8%)	Not assessed directly; better tolerability may imply better usability	Headache, drowsiness more in IV group	Oral ketamine better tolerated; efficacy similar but IV dropout high

Quality Assessment

As shown in Table 2, the risk of bias assessment across the 10 included RCTs was generally favorable, with four studies rated as having an overall low risk of bias, while six studies were rated as having some concerns primarily due to design-related limitations. Most studies demonstrated strong methodological rigor in the randomization process, with all 10 trials achieving a low risk rating in this domain. However, concerns arose in the measurement of outcomes and potential deviations from intended interventions, particularly in open-label designs or subjective-response-dependent studies such as those involving psilocybin. Additionally, selective reporting and missing data were minimal in most cases, although observational or follow-up extensions (e.g., Goodwin et al. [14]) and high dropout rates in pilot trials (e.g., Kumar et al. [20]) introduced moderate concerns. Despite these limitations, the majority of studies maintained sound internal validity, supporting the overall robustness of the evidence base included in this review.

**Table 2:** Quality assessment of each of the selected studies.

Study	Randomization Process	Deviations from Intended Interventions	Missing Outcome Data	Measurement of Outcome	Selective Reporting	Overall Risk of Bias
Glue et al., 2024 [11]	Low	Low	Low	Low	Low	Low risk
Jha et al., 2024 [12]	Low	Some concerns (open-label design)	Low	Some concerns	Low	Some concerns
Papakostas et al., 2024 [13]	Low	Low	Low	Low	Low	Low risk
Goodwin et al., 2025 (5D-ASC) [14]	Low	Some concerns (unblinding risk due to subjective experience)	Low	Some concerns	Some concerns	Some concerns
Smith-Apeldoorn et al., 2024 [15]	Low	Some concerns (open-label extension)	Low	Low	Low	Some concerns
Martin et al., 2024 [16]	Low	Low	Low	Low	Low	Low risk
Morrison et al., 2024 [17]	Low	Low	Low	Low	Low	Low risk
Vieta et al., 2025 [18]	Some concerns (open-label design)	Low	Low	Low	Low	Some concerns
Goodwin et al., 2025 (follow-up) [19]	Some concerns (observational extension)	Low	Some concerns	Some concerns	Some concerns	Some concerns
Kumar et al., 2024 [20]	Some concerns (pilot design, open-label)	Some concerns	Some concerns (high IV dropout)	Low	Low	Some concerns

Discussion

The present review aimed to compare the efficacy and functional outcomes of psychedelic-assisted therapies in the management of TRD. Across the 10 included randomized clinical trials, multiple psychedelic agents, including oral and intravenous ketamine, esketamine nasal spray, and psilocybin, demonstrated significant antidepressant effects. Notably, Glue et al. [11] reported that extended-release oral ketamine tablets significantly reduced MADRS scores by 6.1 points compared to placebo (p = 0.019), with a marked reduction in relapse rates (43% vs. 71%). Similarly, in a pragmatic comparison by Jha et al. [12], intravenous ketamine was found to be more effective than ECT in moderate outpatient TRD, while ECT had an edge in early response among severe inpatient cases. Psilocybin therapy [14] showed strong correlations between the intensity of the psychedelic experience and reductions in depressive symptoms (r = -0.51 to -0.64), suggesting a dose-response relationship tied to subjective experience. Additionally, the follow-up data demonstrated sustained antidepressant effects with 25 mg psilocybin, extending up to a median of 189 days.

In terms of functional outcomes, Vieta et al. [18] provided the most comprehensive evidence, showing that esketamine nasal spray significantly improved functional remission and workplace productivity over 32 weeks compared to quetiapine XR (p = 0.0023 and p = 0.0045, respectively). Cognitive safety was consistently upheld across multiple studies, including those by Martin et al. [16] and Morrison et al. [17], with no evidence of cognitive impairment following repeated ketamine or esketamine use. Oral formulations, as seen in studies by Kumar et al. [20] and Smith-Apeldoorn et al. [15], were generally better tolerated and associated with fewer adverse events compared to intravenous routes. Collectively, these findings affirm that psychedelic-assisted therapies, particularly ketamine-based and psilocybin interventions, offer clinically meaningful benefits in TRD with acceptable safety profiles and emerging evidence for functional improvement. These findings reinforce one of the most clinically significant advantages of ketamine and esketamine - their very rapid onset of antidepressant action, often observable within hours to a few days following administration. This contrasts with conventional antidepressants, which may take several weeks to exert therapeutic effects, and positions ketamine-based therapies, especially valuable in acute depressive episodes or crisis situations in TRD.

The findings of this review underscore the growing clinical viability of psychedelic-assisted therapies as rapid-acting and potentially durable treatments for individuals with TRD - a population historically difficult to manage with conventional antidepressants [21]. The consistent reductions in depressive symptom scores across diverse agents, such as ketamine, esketamine, and psilocybin reflect a robust antidepressant signal, with some therapies such as psilocybin demonstrating effects strongly correlated with the intensity of the psychedelic experience, suggesting that the mechanism may involve not only neurochemical modulation (e.g., N-methyl-D-aspartate (NMDA) antagonism, serotonergic agonism) but also psychological transformation or neuroplasticity [22]. Notably, esketamine nasal spray was associated with functional improvements and enhanced productivity, indicating that these agents may extend benefits beyond symptom control into real-world recovery domains [23]. The durability of psilocybin's effects and the cognitive safety of ketamine-based treatments further reinforce their promise in chronic and refractory cases. Collectively, the results suggest a paradigm shift wherein psychedelics may supplement or, in some cases, supersede traditional pharmacotherapies for TRD, with implications for both clinical practice and public health systems aiming to reduce the burden of chronic depression [24,25].

The results of this review align with previous meta-analyses and systematic reviews that have highlighted the rapid and substantial antidepressant effects of ketamine and psilocybin in TRD. Prior literature has consistently demonstrated that ketamine produces a significant reduction in depressive symptoms within hours to days, a pattern mirrored in studies such as those by Glue et al. [11] and Jha et al. [12], reinforcing its role as a fast-acting intervention. Similarly, the observed correlation between psychedelic intensity and therapeutic response in psilocybin studies is consistent with earlier findings suggesting that the quality of the psychedelic experience is a key mediator of clinical outcome. However, this review adds further depth by incorporating data on functional outcomes and cognitive safety, areas often underrepresented in earlier reports. Unlike some past trials that raised concerns about dissociation or cognitive side effects, recent data, such as from Morrison et al. [17] and Martin et al. [16], show no clinically meaningful cognitive decline, suggesting improved safety with optimized dosing and patient selection. Minor discrepancies remain in the durability of response and optimal routes of administration, especially for oral versus intravenous ketamine, warranting further investigation.

A key strength of this review lies in its focused synthesis of recent high-quality RCTs, offering a contemporary evaluation of psychedelic-assisted therapies with both efficacy and functional outcome lenses. The inclusion of diverse interventions - ranging from ketamine and esketamine to psilocybin - alongside assessments of cognitive safety and productivity outcomes, adds a novel and clinically meaningful dimension often overlooked in previous reviews. However, certain limitations must be acknowledged. Variability in study designs, dosing regimens, follow-up durations, and outcome measures introduces heterogeneity that may limit direct comparability across trials. Additionally, some studies lacked functional or long-term outcome data, and a few were open-label in design, introducing potential performance and detection biases. These factors may have influenced the strength or generalizability of the conclusions and highlight the need for more standardized, head-to-head trials with consistent reporting on both clinical and real-world outcomes.

The findings of this review have significant clinical implications, reinforcing the potential of psychedelic-assisted therapies as viable alternatives or adjuncts to traditional antidepressants in managing TRD. Agents such as esketamine and psilocybin not only demonstrate rapid symptom relief but also show emerging benefits in functional recovery and cognitive safety, making them particularly attractive in real-world settings where quality of life and productivity are key considerations [22]. These results support broader integration of psychedelic-assisted treatments into clinical practice, particularly for patients unresponsive to first- and second-line therapies. On a policy level, this growing evidence base could guide regulatory bodies in expanding access to these therapies through updated clinical guidelines, insurance coverage decisions, and the establishment of specialized treatment protocols that include careful patient selection, psychological support, and monitoring frameworks.

Integrating both functional and efficacy outcomes provides a more comprehensive and patient-centered evaluation of psychedelic-assisted therapies in TRD. While symptom reduction remains a critical measure of treatment success, improvements in daily functioning, work productivity, and cognitive performance are equally vital indicators of meaningful recovery. This review highlights that interventions such as esketamine nasal spray not only reduce depressive symptoms but also lead to significant gains in real-world functioning, as evidenced by improvements in the Sheehan Disability Scale [26] and workplace productivity metrics. Such dual outcomes better reflect the true impact of treatment on patients' lives, informing clinical decision-making that prioritizes not just symptom remission but also quality of life and social reintegration. By adopting this integrative approach, clinicians and researchers can more effectively evaluate and compare interventions, ultimately guiding more holistic and impactful care strategies. Future research should focus on standardizing outcome measures across trials, particularly in assessing functional and cognitive domains, alongside symptom remission, to better capture the holistic impact of psychedelic-assisted therapies. There is a need for longer-term, large-scale comparative studies to evaluate the durability of treatment effects, especially for single-dose agents such as psilocybin. Additionally, research should explore optimal dosing strategies, combination therapies, and real-world implementation models, including home-based or digital-assisted care. Trials incorporating biomarkers, neuroimaging, and patient-reported outcomes could further clarify mechanisms of action and help personalize treatment approaches. Addressing current gaps, such as limited data on diverse populations and long-term safety, will be essential in refining clinical guidelines and ensuring equitable, evidence-based access to these emerging interventions.

## Conclusions

This review affirms the growing clinical relevance of psychedelic-assisted therapies as promising interventions for treatment-resistant depression, offering not only rapid and significant symptom relief but also emerging benefits in functional and cognitive domains. The evidence supports the use of agents such as ketamine, esketamine, and psilocybin as viable alternatives or adjuncts to conventional treatments, particularly in patients who have failed to respond to standard antidepressants. With generally favorable safety profiles and the potential to enhance real-world functioning, these therapies represent a transformative shift in the management of chronic depression. Thus, they warrant continued clinical adoption and rigorous investigation to optimize their integration into psychiatric care.
